# Hemorrhage Following Extraction of a Tooth

**Published:** 1871-12

**Authors:** S. P. Cutler


### ARTICLE IV.

### *Hemorrhage Following Extraction of a Tooth.*

BY S. P. CUTLER, M. D., D. D. S.

A case in March 1871, a young man, a creole about 24 years of age, called for the purpose of having a left lower molar extracted and wished to take gas. I gave him a dose as usual from the beexy, and proceeded to business in a surgical way or rather unsurgical way. My forceps having been selected previously, though not just such a one as I wished, the mouth propped open with a bottle cork with a string tied to the end of it to prevent swallowing—which accident has happened, I adjusted the instrument, in a hurry, as is usually the case when gas is used, and proceeded to pull, and my instrument seeming to loose its hold in some way, and my patient apparently coming to, I hurried matters up rather too rapidly I suppose, and instead of pulling one tooth I pulled two, the right one and a wrong one, the wisdom being the wrong one, which was lifted nearly out of its socket so that I could remove it easily with my fingers, but instead I pushed it down back into its place, although my patient was aware of the true state of the case and left it with me to decide. In some way my forceps' outer beak had slipped off from the tooth and passed between the two teeth while the inner beak remained in position ; this being the condition of things when the teeth gave way, a piece of the alveola between the teeth also having been pulled loose which afterward came away, but not known at the time. After a short period the patient left, after some directions concerning the replaced tooth.

This was about 11 o'clock, and in about three quarters of an hour he returned accompanied by his mother and a sister, the patient spitting up large quantities of blood from the socket. We resorted to astringents, such a persulphate of iron first, and waited for a time, still no abatement of the hemorrhage, the blood flowing copiously. The next thing devised was plugging the sockets with cotton saturated with styptic colloid, this like the first did no good whatever, as the cotton continued to rise up out of the sockets. Next waxed cones were tried for plugging the sockets and the upper teeth closed upon them, still no abatement; several trials were made in this way none of which done any good at all. After this, various astringents were tried all in vain, and by this time some confusion and excitement prevailed, the friends becoming somewhat uneasy as well as ourselves. During the multitudinous trials of various astringents the mouth became perfectly black by the incautious use of Monsel's solution and tannin in succession, without our thinking of converting his mouth into an inkstand at the time, which at first increased the alarm of the family, who by this time had arrived consisting of eight or ten persons in all; the explanation seemed to be satisfactory.

Next thing in the programme was to make a plaster cast of jaw and sockets as a hemorrhagic splint, but all to no purpose.

The blood flowed if anything faster. Next resort was to try a gutta-perch cast and splint, with no better result, astringents being used in conjunction until all the most powerful known had been tried in vain. Darkness came on adding to our confusion. About this time or shortly after dark, the old lady had a regular nervous spasm or fit which increased the fears of our patient still more. Thus matters went on until about 8 o'clock when I began to loose hope and confidence in our ability to staunch the blood at all, as every kind of pressure and all kinds of local remedies had proved a humbug and failure. We then seriously discussed the propriety of the actual cautery. Long before this time the replaced tooth had been removed with the thumb and finger. Things began to look gloomy in the extreme, and what more could be done. One last resort more, I had thought of nitrate of silver but did not have over-confidence in it, as I had sometimes failed with it. I plunged a stick deep down into the sockets alternately as a forlorn hope. The blood ceased almost instantaneously and never bled any at all after that. On the patient attempting to leave the operating chair with help to go to the sofa he fainted from loss of blood, having lost in our estimation at least half a gallon, or perhaps more. Just at the close of our manipulations his family physician arrived, and escorted him home in a carriage to our joy and great relief.

I had begun to think our patient a doomed man. I am now unable to account for the persistent hemorrhage, as there was no undue laceration of the parts. It is not likely that the gas had any agency by its action on the blood. There was no indication of a hemorrhagic diathesis, his temperament being nervo-bilious, the former predominating. The question is, would this hemorrhage have taken place from the extraction of one tooth only and no injury to the bone? I have before had persistent cases of hemorrhage, but not to compare with this one. I can say in confidence that this was the first and only time I ever extracted a wrong tooth during a long time of practice, and this would not have happened without the use of the gas. In hurrying I made a regular *faux pas*, an unpardonable blunder, an irreparable one in the loss of a good tooth to the patient.

I use gas still, but believe it to be a great humbug, one of the greatest of evils so far as dentistry is concerned, or any other anæsthetic used in dentistry. Multiplied thousands of teeth are annually sacraficed by its use that without it might be saved. Gas and cheap rubber plates are the order of the day, and no one can stop the tide. Perhaps none of my readers ever committed any such blunder in their practice. I sincerely hope that none of them ever did or ever will, if so they have my sympathy in full.

---

